# Effects of combining functional exercises with exercise training on daily physical activities and functionality in patients with COPD: a protocol for a randomized clinical trial

**DOI:** 10.1186/s13063-019-3780-y

**Published:** 2019-12-05

**Authors:** Fabiano Francisco de Lima, Carlos Augusto Camillo, Isis Grigoletto, Juliana Souza Uzeloto, Franciele Marques Vanderlei, Dionei Ramos, Ercy Mara Cipulo Ramos

**Affiliations:** 1 Department of Physiotherapy, Postgraduate Program in Physiotherapy, São Paulo State University (UNESP), Rua Roberto Simonsen, No. 305, Presidente Prudente, São Paulo, 19060-900 Brazil; 2 Department of Physiotherapy, Postgraduate Program in Rehabilitation Sciences, State University of Londrina (UEL), Avenida Robert Koch, 60 – Vila Operária, 86038-350 Londrina, Brazil; 3 Department of Rehabilitation Sciences, University Pitágoras UNOPAR, Avenida Paris, 675 – Jardim Piza, 86041-120 Londrina, Brazil

**Keywords:** Chronic obstructive pulmonary disease, Exercise training, Physical activity levels, Follow-up

## Abstract

**Introduction:**

Functional training has been shown to be a viable alternative for the elderly and patients with chronic obstructive pulmonary disease (COPD). However, whether the combination of this type of training with aerobic and resistance training, commonly performed in pulmonary rehabilitation (PR) programs, induces more pronounced effects on daily physical activities and functionality remains unclear. The aims of the study will be to evaluate the short-term and sustained effects of the combination of a functional circuit program with a training program consisting of aerobic and resistance exercise.

**Methods:**

In this randomized controlled trial, patients with COPD will be randomly assigned (1:1:1) to an 8-week training program to follow one of the three *a priori* defined groups: (I) resistance and aerobic and functional exercises, (II) a conventional program including only resistance and aerobic exercises, or (III) a usual care program. Patients will be evaluated before and upon completion of 8 weeks of training regarding physical activity in daily life (PADL) using an activity monitor (accelerometer), activities of daily living (London Chest Activity of Daily Living), functional exercise capacity (6-minute walk test), and muscle strength (dynamometry). Additionally, the sustained effects of the interventions will be evaluated 22 weeks after commencing the study.

**Discussion:**

The inclusion of a protocol of functional physical training in the training conventionally performed by patients with COPD as an alternative to increase PADL and functionality may provide subsidies for the treatment of these patients, representing an advance and impacting on the physical training of patients with COPD.

**Trial registration:**

Brazilian Clinical Trials Registry (ReBEC) ID: RBR-3zmh3r. Registered: March 7, 2018.

## Introduction

Despite its primarily respiratory character, chronic obstructive pulmonary disease (COPD) also presents extrapulmonary consequences, including the reduction in functional exercise capacity and musculoskeletal function (i.e., strength and peripheral muscular resistance) [1, 2]. It is known that, to avoid the symptoms of the disease, these patients are less active when compared with healthy individuals of the same age group, presenting less time in physical activity, at a lower intensity and number of movements per day [3–8]. Lower levels of physical activity in daily life (PADL) are associated with a higher risk of hospitalizations and worse prognosis in COPD [9, 10], besides contributing to the progression of the disease [11].

As an attempt to counteract these consequences, pulmonary rehabilitation (PR) programs including physical training have been proposed as a key element in the treatment of COPD [12, 13]. Physical training is associated with an improvement in the functional exercise capacity and a reduction in dyspnea in these individuals [14, 15]. Resistance and aerobic training are among the exercises commonly proposed for these patients [12, 13, 16]. The combination of these modalities has been shown to be the best form of training for these individuals [17] as well as being recommended by international guidelines for the clinical treatment of these patients in PR programs [13, 18]. Despite the functional gains obtained in PR programs, the evidence for increased PADL in patients with COPD is still contradictory and inconsistent [19], especially in short-term PR programs [20–24].

Functional physical training has been shown to be an effective alternative for improving mobility, functionality, and performance in the activities of daily living (ADLs) of older adults [25–27]. This type of training includes muscle work involving coordinated patterns of multi-joint movements and dynamic tasks in order to improve functionality. Studies demonstrate positive functional effects of this type of exercise for patients with COPD [22, 28].

Sewell et al. [28] demonstrated improvement in PADL and ADL performance after a short-term training program combining functional exercises with aerobic and home training in patients with COPD. Despite the benefits obtained, the authors did not evaluate whether the responses were maintained for a longer period of time than the program period and did not include the progressive resistance training, an essential component for these patients. It is still unknown whether the addition of a functional circuit training (structured on the basis of the limitations during ADLs) to a conventional combined aerobic and resistance training program, commonly performed and well established in PR programs, can improve PADL, ADL performance, and functionality of patients with COPD in the short term. In addition, it is unknown whether possible functional and PADL gains obtained in this type of training are maintained during a 3-month follow-up.

This randomized clinical superiority trial aims to evaluate the effects of the addition of a circuit of functional physical training to an 8-week combined training (aerobic and resistance) on PADL, ADL performance, functional exercise capacity, and peripheral muscle strength of patients with COPD. In addition, the effects of the interventions will be re-evaluated 3 months after the training completion. It is hypothesized that the proposed functional training will lead to behavioral changes, influencing the improvement in the performance of ADLs and, consequently, PADL, accompanied by functional improvement in patients with COPD.

## Methods

### Study design

**Fig. 1:**
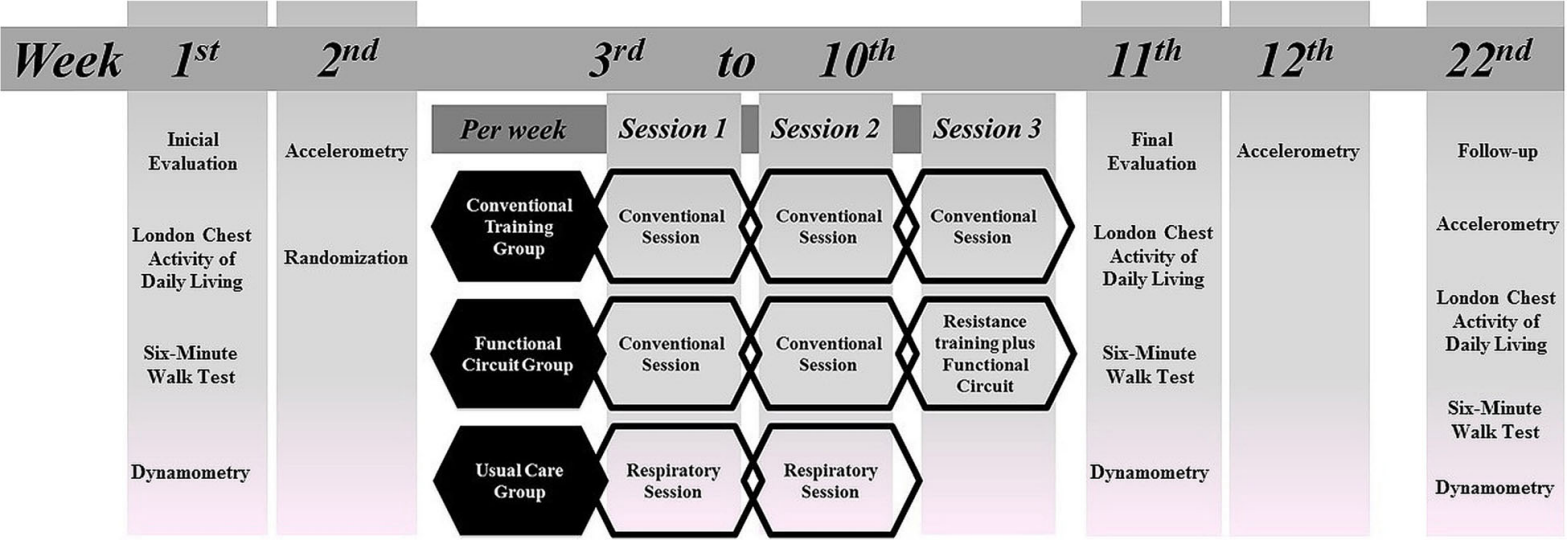
Experimental design of the study

A randomized clinical trial with follow-up will be conducted at the Center for Studies and Care in Physical Therapy and Rehabilitation of the Universidade Estadual Paulista (FCT/UNESP) in Presidente Prudente, Brazil. The study protocol was developed following the Standard Protocol Items: Recommendations for Interventional Trials (SPIRIT) 2013 Checklist guidelines (Additional file 1) [29]. The trial was registered at www.ensaiosclinicos.gov.br (ID: RBR-3zmh3r). The study design is shown in Fig. 1.

Initially, participants will participate in an assessment process over 2 weeks on non-consecutive days. The first day of evaluation will consist of anamnesis, obtaining personal identification data and investigation of pre-existing comorbidities, and performing spirometry to confirm the diagnosis of COPD by means of a portable spirometer MIR-Spirobank version 3.6 (MIR - Medical International Research, Roma, Italy). The interpretation will be in accordance with the American Thoracic Society (ATS) and European Respiratory Society (ERS) [30], and values of normality will be relative to the Brazilian population [31]. Limitations of ADLs will be evaluated by the London Chest Activity of Daily Living (LCADL) scale [32, 33]. It should be emphasized that the questionnaire will be applied through an interview, always by the same evaluator, who will carry out prior training to avoid possible biases in the interpretation of questions and answers. On the second day, a functional capacity evaluation will be performed using the 6-minute walk test (6MWT). On the third day of evaluations, the measurement of peripheral muscle strength will be performed by means of a digital dynamometer. Patients will receive a physical activity monitor (accelerometer) which will be used during 7 days before the beginning of the training period, together with the basic guidelines for using the equipment. After the initial evaluation period, the training phase will begin. Conventional sessions will include resistance training and aerobic training. Resistance training plus functional circuit will include resistance training and performing a functional training circuit. Respiratory sessions will include only respiratory physiotherapy techniques.

After the end of the training period (8 weeks), all the evaluations described above, except for the spirometry, will be repeated. Finally, a follow-up will be performed 3 months after the end of the training period, evaluating the PADL, limitations in the performance of ADLs, functional exercise capacity, and muscle strength.

### Participants

For this study, 75 patients with COPD from the municipality of Presidente Prudente and the region will be recruited through telephone contact and the distribution of leaflets and medical referrals. The following inclusion criteria will be considered: (1) patients with COPD diagnosed according to the Global Initiative for Chronic Obstructive Lung Disease (GOLD) [34], (2) clinically stable patients without exacerbations or changes in medications for at least 30 days, (3) patients who do not use long-term oxygen therapy at home, (4) patients without pathological conditions that prevent the performance of physical activity, (5) absence of severe or unstable heart disease, and (6) do not participate in another structured exercise program. The study was approved by the research ethics committee of the FCT/UNESP in Presidente Prudente, Brazil (#77909317.2.0000.5402).

### Analysis of the population

Exclusion criteria are complications that prevent the continuity of the training protocol as well as low adhesion rate to the training protocol (below 75% of all sessions) [35]. In the event of two consecutive absences from training sessions, patients will be contacted by telephone to confirm the reason. An intention-to-treat analysis will be performed using the patient’s most recent assessment in case of withdrawals or absence of data. In case of possible injuries occurring during the intervention period, the individuals will be referred for appropriate treatment.

### Randomization

The sample will be randomly allocated into three groups: functional circuit training group (FTG), conventional training group (CTG), and usual care group (UCG). The randomized allocation sequence will be performed by a researcher who will not be involved in the recruitment, evaluation, or training of patients, and an online platform (www.sealedenvelope.com) and concealed brown envelopes will be used. The randomization process will be carried out in blocks defined *a priori*. Evaluators will be blind as to the allocation of participants to interventions. The training will be supervised by trained physiotherapists not involved in the randomization process or evaluations.

## Procedures

### Interventions

The physical exercise program will take place over 8 weeks with a frequency of three weekly sessions of about 60 min each, totaling 24 training sessions. The sessions will begin with dynamic general stretching, after which the aerobic training will be carried out on a treadmill with a duration of 30 min, followed by the resistance training of upper limbs and lower limbs. These dynamics will occur for both the FTG and CTG, except for the third weekly session in which the FTG will perform functional training in circuit format. At the beginning, during, and at the end of the sessions, vital signs will be checked. The UCG will perform only usual care involving respiratory physiotherapy sessions that include inhalation therapy, pulmonary deflation techniques, diaphragmatic awareness, and inspiratory muscle exercise.

#### Resistance training

For the training prescription, a 1-repetition maximum (1RM) test will be performed, according to a previously described protocol [36, 37], of the following muscle groups: elbow flexors and knee extensors and flexors. The resistance training will be performed using weight machines: extensor chair and flexor chair (Ipiranga® - Brazil) for lower limbs and simple pulley equipment (Ipiranga® - Brazil) for upper limbs. The intensity of training will follow the protocol recommended for patients with COPD [38, 39], 60 to 80% of the 1RM test, with three sets of 10 repetitions, and 2-min intervals between sets. The load increase will be performed every four stimuli (sessions) with a 5% increase in the intensity of the 1RM test until reaching 80%. The trained muscle groups will be the same as previously described in the 1RM test.

#### Aerobic training

The aerobic training will be performed on an ergometric treadmill and will begin with an intensity of 80% of the average speed reached in the 6MWT [40]. In addition, the increase in aerobic training intensity will be performed on the basis of the subjective sensation of dyspnea of the individuals, measured by the Borg scale [38]. Thus, when the individual reports a dyspnea sensation with values between 4 and 6 on the Borg scale, the intensity will be maintained, this being considered an adequate training intensity [38, 41]; however, when the intensity is less than 4, there will be a 5% increase in training intensity.

#### Functional circuit training

In order to maintain the same increment dynamics of resistance training in both training groups (FTG and CTG), the session that will include circuit format functional training in the FTG will be divided into two stages: functional circuit and resistance training (elbow flexion, knee extension, and flexion). The circuit training will be composed of 12 exercises (stations) that will simulate ADLs, elaborated on the basis of previous identification (telephone interview) of the main limitations for performing ADLs, reported by patients with COPD from the database of the care center where the study will be conducted.

Each exercise will last 2 min and 30 s, so the total duration of the functional circuit will be 30 min, as performed in aerobic training. Following the same method of aerobic training, the increment in the training intensity will be performed on the basis of the subjective sensation of dyspnea of the individuals, measured using the Borg scale [38, 41]. Thus, when the individual reports a dyspnea sensation with values between 4 and 6 on the Borg scale, the intensity will be maintained, this being considered an adequate training intensity [38, 41]; however, when the intensity is less than 4, the training intensity will be increased (that is, an increase in the speed/number of repetitions of the exercises until the 8 weeks of training is completed). A pilot session was conducted with patients with COPD to evaluate the viability and execution time of functional training in circuit format. There were no problems in the execution and time of the exercises and these were shown to be feasible by the patients.

**Fig. 2:**
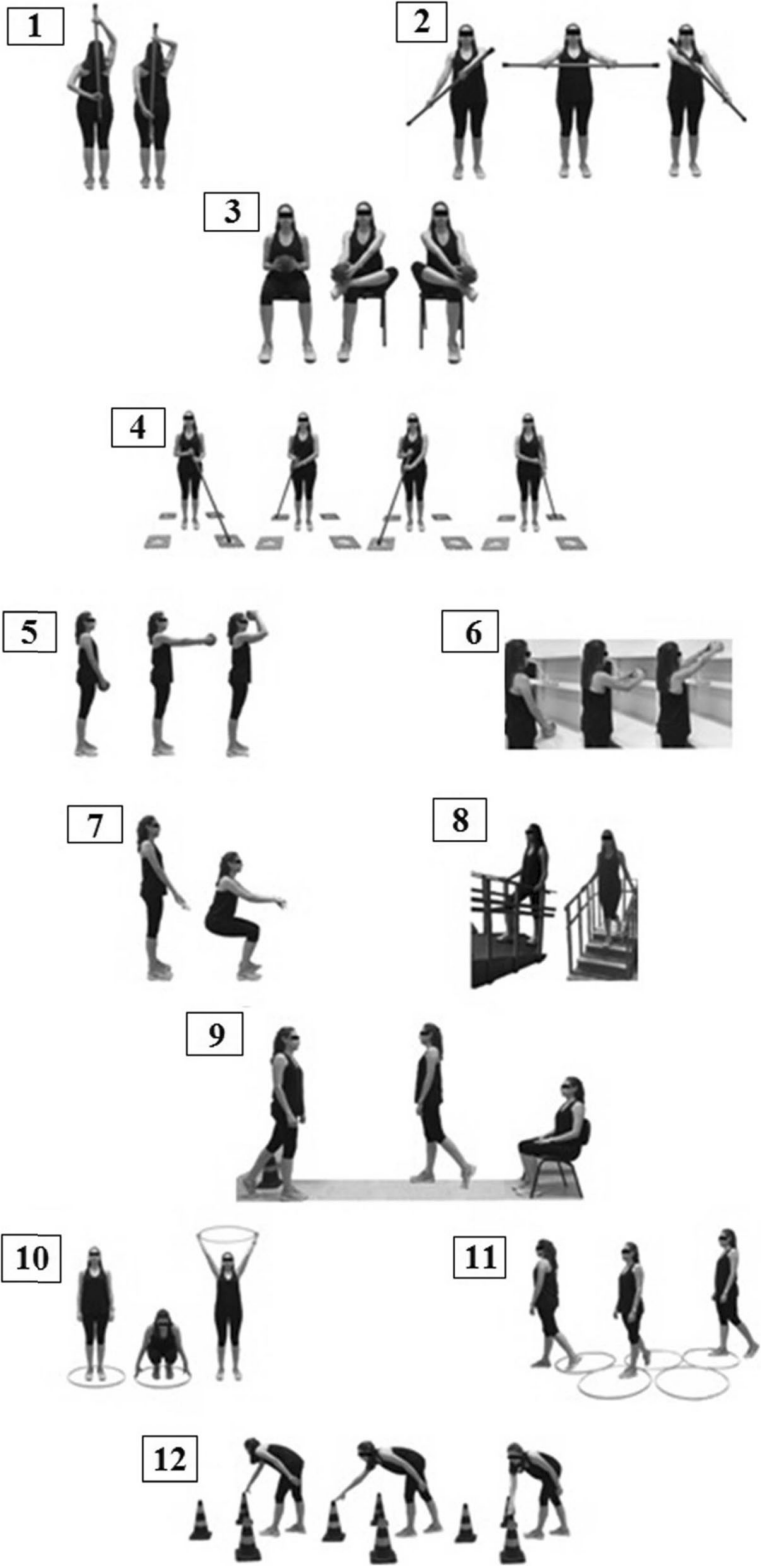
Functional circuit exercises

The functional circuit will include the exercises/stations described below and illustrated in Fig. 2:

Exercise 1: Simulate drying the back: pass the stick behind the back (as if drying the back with a towel) and change hands every five movements.

Exercise 2: Simulate sweeping the floor: With shoulder flexion at about 75°, hold the stick with the forearm pronated and perform a movement similar to “rowing”.

Exercise 3: Simulate tying shoes: Sitting in a chair with 90° elbow flexion, hold a ball with the hands, then perform knee flexion, touching the lateral malleolus on the contralateral knee, with the ball touching the medial malleolus.

Exercise 4: Simulate passing a squeegee on the floor: Holding a stick (the stick should touch the ground) with the hands, move the stick anteriorly to the left and then to the right and then repeat on the contralateral side.

Exercise 5: Simulate bath movements to wash the hair: Holding a small ball in the hands, perform simultaneous movements: 90° shoulder flexion, 90° elbow flexion, and touch the ball on the head.

Exercise 6: Simulate picking up objects in high and low places: In front of a bookcase in the orthostatic position, pick up a ball from a high shelf (head level) and then put it on two lower shelves (chest level and waist level).

Exercise 7: Simulate squatting: Squat holding a fixed bar.

Exercise 8: Simulate walking on uneven ground using ramps and stairs: Rise and descend steps/ramp.

Exercise 9: Simulate standing up and sitting in a chair: Get up from the chair, walk a short distance (3 m), and sit in the chair again.

Exercise 10: Simulate changing clothes: With a hula hoop on the ground, step with both feet inside the circle, crouch to pick up the hula hoop with both hands, rise up holding the hula hoop, and perform shoulder flexion by lifting the hula hoop so that it runs all over the body of the individual; reverse the movement with the same steps, ending by placing it on the floor again.

Exercise 11: Simulate the avoidance of obstacles during gait: With five hula hoops on the ground, walk among the hula hoops.

Exercise 12: Simulate picking up objects: In front of three small cones on the floor, perform trunk flexion and touch the tip of one of the cones and return to the starting position and repeat the movement until having touched all the cones. When finishing the three movements, perform them with the other hand (Fig. 2).

### Participant timeline

**Fig. 3:**
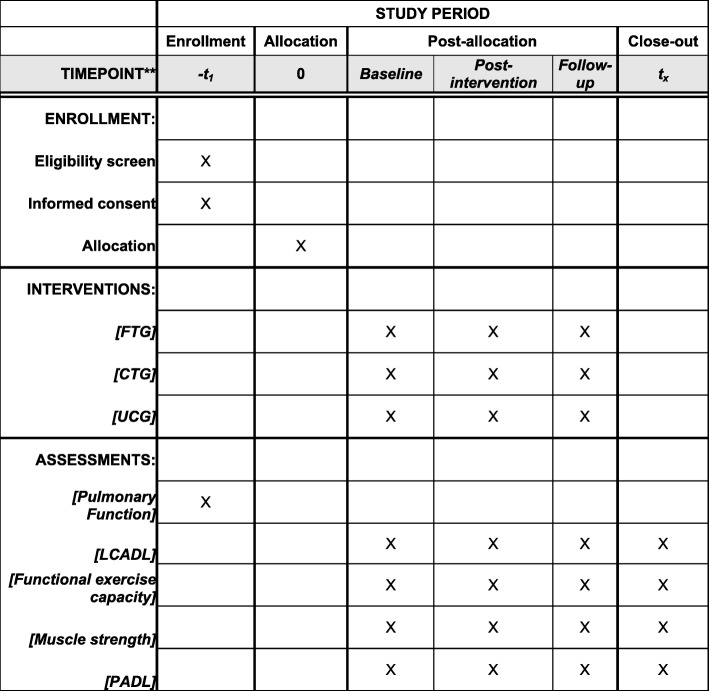
Content for the schedule of enrollment, interventions, and assessments. *Abbreviations*: *CTG* conventional training group, *FTG* functional circuit training group, *LCADL* London Chest Activity of Daily Living, *PADL* physical activity in daily life, *UCG* usual care group.

The time schedule of enrollment, interventions, and assessments is outlined in Fig. 3. Recruitment of study participants began in July 2018.

## Results

The primary outcome will be the PADL assessment using accelerometry. Secondary outcomes are limitations on performance in ADLs, functional exercise capacity, and peripheral muscle strength. The evaluations will be carried out by the same evaluators at all described moments.

### Primary outcome

The PADL will be used as the primary outcome of the study, measured by step counts days and time spent on activities, using an accelerometer-type movement sensor, ActiGraph GT3X (ActiGraph LLC, Pensacola, FL, USA), which measures and records acceleration variations with magnitudes ranging from approximately 0.05 to 2.5 G (g = 9.8 m/s [2]) within a frequency range of 0.25 to 2.5 Hz. Each sample of counts will be summarized over a specific time interval of 60 s, called an epoch. Accelerometers will be placed on the waist immediately below the umbilical scar, and individuals will wear the equipment for 7 days. The accelerometer will be removed during the nighttime sleep period and when the participant has contact with water (personal hygiene or water activities). Participants will wear the device before and after the intervention periods and soon after the 3-month post-intervention follow-up. For analysis of the data, specific *software* will be used (ActiLife5 – Data Analysis Software by ActiGraph).

### Secondary outcomes

The study will have three evaluations of secondary outcomes: limitations during ADL performance, functional exercise capacity, and peripheral muscle strength. These outcomes will be evaluated before and after the intervention period as well as after the post-intervention follow-up period of 3 months.

#### Limitations during activities of daily living

Limitations during the performance of ADLs will be evaluated by the LCADL scale, developed by Garrod et al. (2000) [42], translated to Portuguese, and validated for use in COPD in Brazilian patients [32, 33]. This scale contains 15 items divided into four domains: personal care (four items), domestic (six items), physical activity (two items), and leisure (three items) [33]. The total score can vary from 0 to 75 points, and the higher the score, the greater the limitation in ADLs [32].

#### Functional exercise capacity

Functional exercise capacity will be evaluated through the 6MWT, performed in accordance with the guidelines established by the ATS [43].

#### Peripheral muscle strength

Measurement of muscle strength will be performed in the dominant member by using a Force Gauge digital dynamometer, (Instrutherm - model DD-300), and the results will be expressed in newtons. The patient will be instructed to perform the movements of elbow flexion and knee flexion and extension, resisted by a steel cable coupled to the dynamometer. The measurement will be repeated five times with an interval of 1 min between attempts, and the highest value among the three closest measurements will be recorded [44].

### Sample size calculation

This is a superiority trial in which sample size determination was performed through prior study [45] information based on the primary PADL variable. With the expectation of an increase in the number of steps of 45% (approximately 2260 steps) in the FTG compared with the CTG and UTG (both with no expected increase), using a standard deviation of 2603 steps, and loss at follow-up of 20% of individuals (values commonly found in the study population) [44], 25 individuals will be required in each of the three groups to obtain a power of 80%, adopting an α of 5%.

### Statistical analysis

The statistical program SPSS 22.0 (IBM, Armonk, NY, USA) will be used. The data will be submitted to the normality test of Kolmogorov–Smirnov, and if they present normal distribution, the descriptive variables will be expressed as mean and standard deviation; if they do not fit the Gaussian distribution model, data will be presented as median and interquartile range. Mauchly’s sphericity test will be performed. Once the sphericity is assumed, two-way analysis of variance (ANOVA) will be performed to evaluate the possible intra- and inter-group differences (FTG, CTG, and UCG) at pre-intervention, post-intervention, and post-*follow-up*. The Tukey post-hoc will be used to identify the specific differences in the variables in which the F values found are higher than the established statistical significance criterion. To evaluate the effect size between interventions, Cohen’s d test will be used. The level of significance adopted will be 5%. Data integrity will be monitored by regularly scrutinizing data files for omissions and errors. Participants will be given an anonymous study ID to protect confidentiality, and only study investigators will have access to the final trial data set.

## Discussion

### Potential impact and significance of the study

The present proposal was developed through extensive literature research on this topic. Through the interpretation of the results found in several studies, it was possible to develop this concise and structured training protocol. The use of this protocol in the treatment of COPD will confirm whether the addition of functional physical training to a conventional training will promote positive effects on PADL and functionality and whether there will be maintenance of possible gains at the 3-month post-training follow-up.

### Strengths and limitations of the study

This study presents as a strong point the follow-up 3 months after training as this allows evaluation of whether the possible changes resulting from the protocol will be maintained. The assessment of PADL via accelerometers as primary outcome is a strong point as it is an objective measurement and does not suffer influence of personnel involved in the study. Another strong point that can be considered is the fact of this being a randomized clinical trial, which makes the study more reliable. The fact that the study is performed in only one center is considered a limitation, as is the inability to blind the therapists and patients to the training protocol.

### Contribution and clinical applicability

The results obtained in the proposed protocol may provide subsidies via publication for the implementation and insertion of a physical training circuit in the rehabilitation of patients with COPD. In addition, the proposed functional exercises are easily applicable and simulate ADLs not requiring specific equipment and therefore can be implemented in several PR centers.

### Trial status

Protocol number: RBR-3zmh3r. Recruiting: Study start date: March 7, 2018. Study completion date: December 2019.

## Additional file

**Additional file 1.** Standard Protocol Items: Recommendations for Interventional Trials (SPIRIT) 2013 Checklist: Recommended items to address in a clinical trial protocol and related documents.

## Data Availability

Not applicable.

## Abbreviations

1RM: 1-repetition maximum
6MWT: 6-minute walk test
ADLs: Activities of daily living
ATS: American Thoracic Society
COPD: Chronic obstructive pulmonary disease
CTG: Conventional training group
FCT/UNESP: Universidade Estadual Paulista
FTG: Functional circuit training group
LCADL: London Chest Activity of Daily Living
PADL: Physical activity in daily life
PR: Pulmonary rehabilitation
UCG: Usual care group
